# Novel, Real-Time Cell Analysis for Measuring Viral Cytopathogenesis and the Efficacy of Neutralizing Antibodies to the 2009 Influenza A (H1N1) Virus

**DOI:** 10.1371/journal.pone.0031965

**Published:** 2012-02-20

**Authors:** Di Tian, Wanju Zhang, Jing He, Yi Liu, Zhigang Song, Zhitong Zhou, Min Zheng, Yunwen Hu

**Affiliations:** 1 Pathogen Diagnosis and Biosafety Department, Shanghai Public Health Clinical Center Affiliated to Fudan University, Shanghai, China; 2 State Key Laboratory of Infectious Disease Diagnostic and Treatment, First Affiliated Hospital, School of Medicine, Zhejiang University, Hangzhou, China; Centers for Disease Control and Prevention, United States of America

## Abstract

A novel electronic cell sensor array technology, the real-time cell analysis (RTCA) system, was developed to monitor cell events. Unlike the conventional methods labeling the target cells with fluorescence, luminescence, or light absorption, the RTCA system allows for label-free detection of cell processes directly without the incorporation of labels. Here, we used this new format to measure the cytopathic effect (CPE) of the 2009 influenza A (H1N1) virus and the efficacy of neutralizing antibodies in human sera to this virus. The real-time dynamic monitoring of CPE was performed on MDCK cell cultures infected with the H1N1 virus, ranging from 5.50×10^2^ to 5.50×10^7^ copies/mL. The resulting CPE kinetic curves were automatically recorded and were both time and viral load dependent. The CPE kinetics were also distinguishable between different H1N1 stains, as the onset of CPE induced by the A/Shanghai/37T/2009 H1N1 virus was earlier than that of the A/Shanghai/143T/2009 H1N1 virus. Furthermore, inhibition of H1N1 virus-induced CPE in the presence of human specific anti-sera was detected and quantified using the RTCA system. Antibody titers determined using this new neutralization test correlated well with those obtained independently via the standard hemagglutination inhibition test. Taken together, this new CPE assay format provided label-free and high-throughput measurement of viral growth and the effect of neutralizing antibodies, illustrating its potential in influenza vaccine studies.

## Introduction

With the 2009 global outbreak of pandemic influenza, large-scale vaccination against the new strain of influenza A virus subtype H1N1 was employed to prevent its spread in China, and numerous clinical trials were initiated to estimate the antibody responses elicited with the 2009 influenza A (H1N1) virus vaccine [1], [2], [3]. However, there is still an urgent need for safer and more convenient approaches to monitor influenza stains that may cause pandemic influenza with significant levels of morbidity and mortality. As such, high throughput neutralization assays are required to quantify neutralizing antibodies for serodiagnosis during influenza infection as well as for screening candidate vaccines. The traditional measurement of influenza virus neutralizing antibodies involves hemagglutination, immunological procedures, or various methods for cytopathogenic effect (CPE) identification, such as microscopic, colorimetric, and fluorometric assays [4], [5], [6], [7]. Nevertheless, these methods are somewhat labor-intensive, which may increase the risk of aerosol generation [6], [7], [8].

Since 2004, a real-time cell analysis (RTCA) system based on microelectronic biosensor technology was developed to monitor cell events. The electrode impedance, which is displayed as cell index (CI) values, is measured to characterize cellular status across the electronic cell sensor array integrated on the bottom of the electronic microtiter tissue culture plates (E-Plates) [9], [10]. Due to the the novel electronic cell sensor array technology, the RTCA system can provide dynamic monitoring of nearly all of the cells present in the wells without the label molecules. By this means, a wide range of noninvasive, label-free cell-based assays can be performed, such as monitoring of cytotoxicity and cell death, stem cell proliferation, RNA interference, virus-mediated cytopathogenicity, etc. [11], [12], [13], [14]. Here, we report the use of the RTCA system as a tool for quantifying 2009 H1N1 virus-induced CPE in Madin-Darby canine kidney (MDCK) cells in real time. As a proof of concept, this format was used to measure neutralizing antibodies in pre- and post-immune serum from 21 healthy individuals who had received the 2009 H1N1 vaccination; the data were then compared with those obtained by hemagglutination inhibition tests.

## Materials and Methods

### Cells

MDCK cells (ATCC, Manassas, VA, USA) were maintained in complete DMEM supplemented with 10% FBS, 100 U/mL penicillin, and 100 µg/mL streptomycin (Invitrogen, Carlsbad, CA, USA) at 37°C with 5% CO^2^. For the virus culture, serum free DMEM (SFM) supplemented with antibiotics and TPCK-trypsin (Sigma, St. Louis, MO, USA) was used.

### Viruses

The following influenza virus strains were used in this study: A/Shanghai/37T/2009 (H1N1) (SH37T) (GenBank Accession No. GQ253489–GQ253496) and A/Shanghai/143T/2009 (H1N1) (SH143T) (GenBank Accession No. GQ340061–GQ340064, GQ411905–GQ411907 and GQ411909). SH37T and SH143T were originally isolated from a 30-year-old and a 24-year-old male patient hospitalized respectively in late-May and early-June 2009 at Shanghai Public Health Clinical Center (SHAPHC), China. The two patients were both imported cases which respectively from Australia and France, presenting with fever, rhinorrhea, sore throat and cough. Viruses were grown and titrated in MDCK cells at 35°C with 5% CO_2_.

### Human sera

The 2009 influenza A (H1N1) vaccine (inactivated split virion) was produced by the Shanghai Institute of Biological Products, contained the WHO recommended vaccine virus A/California/07/2009 NYMC X-179A, and was issued by the U.S. Centre for Disease Control and Prevention [15], [16]. The vaccine was administered intramuscularly in the upper arm. A total of 21 pre- and post-vaccination serum triples were collected from healthy adult subjects. Pre-vaccination sera were obtained before vaccination (S0 samples). Post-vaccination sera were obtained 7 and 21 days later after vaccination (S1 and S2 samples, respectively). Serum samples were stored at −80°C until required and were heat-inactivated at 56°C for 30 minutes before use.

### Ethics Statement

Written informed consent was obtained from all subjects. The subjects all confirmed that they understood the study procedures. The overall study was reviewed and approved by the Ethics Committee of SHAPHC.

### Real-time PCR

Viral nucleic acid was extracted using a High Pure Viral Nucleic Acid Kit (Roche Applied Sciences, Indianapolis, IN, USA) according to the manufacturer's instructions. Purified viral nucleic acid templates were amplified with real-time PCR using the Diagnostic Kit for 2009 Influenza A/H1N1 Virus RNA (KHB, Shanghai, China) in a Mastercycler ep realplex 2.2 real-time PCR system (Eppendorf, Hamburg, Germany) according to the manufacturer's instructions.

### Cell proliferation assays

The RTCA system (xCELLigence; Roche Applied Sciences, Indianapolis, IN, USA) is comprised of four components: an electronic sensor analyzer, a device station, E-Plates (E-Plate 96), and a control unit with applied software. The device station was located inside a tissue culture incubator and could be switched to any one of the wells of the E-Plate for impedance measurement using the electronic sensor analyzer. Under control of the control unit software, the CI values are then derived from the electronic impedances and graphically represented.

For real-time cell proliferation assays, 50 µL of cell culture media was added to each well of a 96-well E-Plate to obtain background readings. Serial two-fold MDCK dilutions from 4.0×10^4^ to 2.5×10^3^ cells/well in 100 µL of media were then seeded into the E-Plate. The E-Plates containing cells were then incubated for 30 minutes at room temperature and placed on the RTCA SP/MP station located in a cell culture incubator. The CI values were measured automatically every 15 minutes.

To determine the optimal TPCK-trypsin concentration, a cell proliferation assay in SFM containing TPCK-trypsin was performed. When the cells had reached confluence, the E-Plate was removed from the RTCA station and washed twice with SFM. A total of 150 µL of SFM containing TPCK-trypsin at a final concentration of 10–0.31 µg/mL was added. The E-Plate was immediately placed back in the station and CI measurements were subsequently resumed.

### Real-time monitoring of virus-induced cytopathogenicity

A total of 1.0×10^4^ MDCK cells/well was seeded into each well of an E-Plate. One of two H1N1 strains, SH37T or SH143T, was 10-fold diluted (10^−1^ to 10^−6^) in SFM containing 2.5 µg/mL TPCK-trypsin. Both strains were quantitated by real-time PCR and adjusted to an identical viral load of 5.50×10^8^ copies/mL before dilution. When the CI reached stationary phase after seeding, the cells were washed with SFM and then infected with 150 µL of the virus series. Wells were mock infected with only medium as negative controls. After reloading the E-Plate onto the station, the CI values were measured every 15 minutes for up to 190 hours. Additionally, a colorimetric assay using the cell proliferation reagent WST-1 (Water soluble tetrazolium salts) (Roche Applied Sciences, Indianapolis, IN, USA) was also performed to determine H1N1-induced CPE and titrate the virus as described previously [6], [17]. The assay was based on the cleavage of tetrazolium to formazan salts by mitochondrial dehydrogenases of metabolically active cells, resulting in the color change of the well. Thus the formed formaza dye was quantified spectrophotometrically and correlated directly to the cell viability. The ratio of viral load per 50% tissue culture infectious dose (TCID50) was calculated to determine the number of units required to produce the specific endpoint [18].

### Neutralization test (NT) based on the RTCA system

Dilutions of the H1N1 viruses and the test sera were prepared in SFM with TPCK-trypsin to a final concentration of 2.5 µg/mL. The test sera, serially two-fold diluted starting from a dilution of 1∶10, were mixed with an equal volume of medium containing approximately 100 TCID50 of the H1N1 virus. After incubation at 35°C for 60 min, 150 µL of the serum-virus mixture series was transferred to triplicate monolayers of MDCK cells in the E-Plates. Wells containing virus dilutions in the absence of test sera and wells containing only MDCK cells in SFM were included on each assay plate as controls. H1N1-induced CPE was then monitored using the RTCA system (as described above) over 72 hours. The neutralizing titer was defined as the reciprocal of the lowest concentration of serum at which the infectivity of 100 TCID50 of H1N1 was completely neutralized in all the MDCK wells.

### Hemagglutination inhibition (HI) test

The HI test was performed according to standard procedures [19]. The test sera were treated with receptor destroying enzyme (RDE) (Sigma, St. Louis, MO, USA) to inactivate nonspecific inhibitors. The sera were then two-fold diluted starting from a dilution of 1∶10. A 1.5% suspension of guinea pig erythrocytes in PBS and four hemagglutinating units of influenza antigen were used.

### Statistical analysis

Statistical analysis was performed using SPSS v17.0 (SPSS, Chicago, IL, USA) and GraphPad Prism v5.0 (GraphPad Software, La Jolla, CA, USA) software. Geometric mean titers (GMTs) were calculated by assigning a titer of 5 to the samples HI and NT titers below the lower limit of detection (titer <10). The Wilcoxon signed rank test was used to calculate *p* values when comparing GMTs between paired data. *p* values of less than 0.05 were regarded as statistically significant. Spearman's correlation coefficient was used to calculate the correlation between the NT and HI test results. *R* values of more than 0.5 were regarded as closely related.

## Results

### Optimization of MDCK cell numbers and trypsin concentration

**Figure 1:**
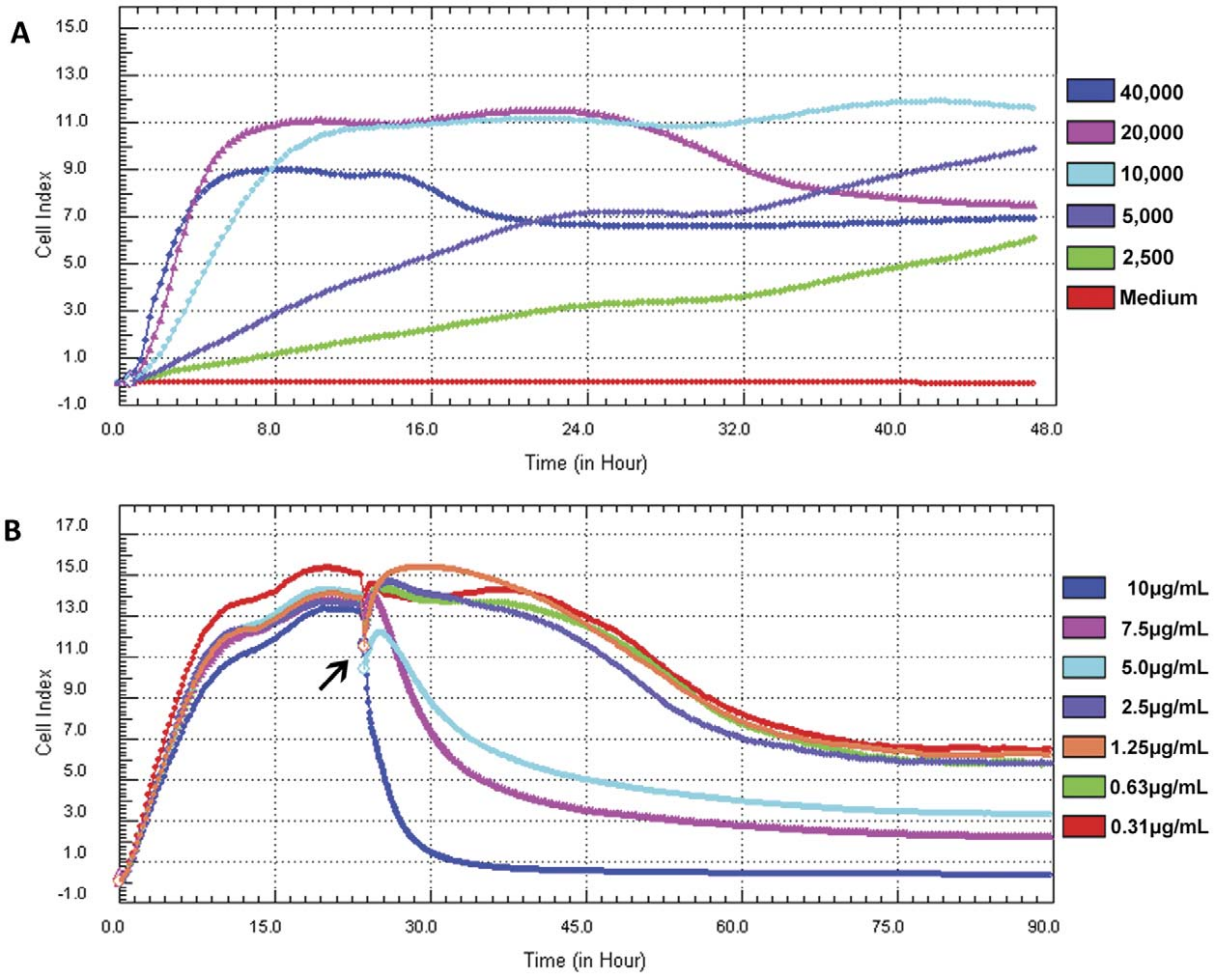
Dynamic monitoring of MDCK cell proliferation. MDCK cells were seeded in the E-Plates and continuously monitored by measuring the CI to identify the appropriate cell numbers, TPCK-trypsin concentration, and the time point for addition of virus (i.e., during growth or early stationary phase). A) Proliferation curves of MDCK cells in cell culture media. B) Proliferation curves of MDCK cells in SFM with TPCK-trypsin. At the indicated time point the cells were inoculated with different concentrations of virus and TPCK-trypsin.

To identify the most suitable MDCK cell numbers, a series of MDCK cell proliferation curves were performed (Figure 1A). Then the level of 1.0×10^4^ cells/well was selected, for which reached the highest CI value during the grow process. As the cells were in the growth phase or the early stationary phase at 16 to 24 hours after seeding, this period of time was suitable for virus infection.

Since infection of the influenza virus into MDCK cells *in vitro* is promoted by the addition of trypsin to the medium, the TPCK-trypsin concentration should be optimized. The cell proliferation of 1.0×10^4^ MDCK cells/well under different doses of TPCK-trypsin (Figure 1B) was performed, and the highest TPCK-trypsin concentration that minimally influenced MDCK cell proliferation was determined to be 2.5 µg/mL.

### 2009 H1N1 virus cytopathogenicity profile

**Figure 2:**
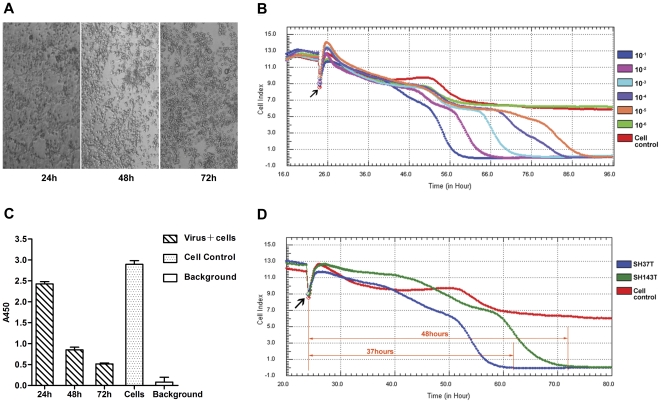
Real-time monitoring of pH1N1 virus-mediated cytopathogenicity. A) The CPE on MDCK cells infected with SH37T (1∶10 diluted) at 24, 48, and 72 hours post-infection. B) Real-time monitoring of the CPE on MDCK cells infected with SH37T using the RTCA system. The curve was an average of four independent replicate wells. At the indicated time point the cells were inoculated with different dilutions of virus. C) The colorimetric assays for the CPE on MDCK cells infected with SH37T (1∶10 diluted). OD values at A450 obtained at 24, 48, and 72 hours post-infection are shown. Error bars indicate standard deviation. D) Difference in CPE kinetic patterns between SH37T and SH143T with the same infectious vial load of 5.50×10^7^ copies/mL. The plotted lines indicate the different time points when the CI declined to 0 between stains.

Based on the dynamic monitoring of cell proliferation, 1.0×10^4^ MDCK cells in stationary phase were infected with SH37T or SH143T at the same viral load of 5.50×10^8^ copies/mL after seeding. In the meantime, the presence of CPE was visualized by microscopic examination (Figure 2A). A decline in CI reflected cell death as a consequence of H1N1 virus replication (Figure 2B), while mock-infected cells continued to grow. Parallel WST-1 colorimetric assays were performed to ascertain whether the CIs correlate with CPE. As shown in Figure 2C, the absorbance values representing the viable cell population were decreased due to CPE. These results demonstrated the accurate monitoring of H1N1-induced CPE using the RTCA system. With the virus load decreased an order of magnitude, the initial CPE was delayed by about 8 hours, illustrating the clear correlation between the onset of CPE and the infectious virus load.

Interestingly, although using the same viral load, cells infected with the different viral strains exhibited different onset times for CPE and complete cell death. For example, for the 10^−1^ dilutions, complete death occurred at 37 hours post-infection for SH37T-infected cells and at 48 hours for SH143T-infected cells (Figure 2D). As for the colorimetric TCID50 test, the ratio of viral load per TCID50 of SH37T was less than that of SH143T (Table 1), which confirmed the results of the RTCA tests. This demonstrated that the RTCA test could not only quantify H1N1 viruses-induced CPE, but it also reflected the different infectivity of the distinct strains.

**Table 1:** TCID50, viral load, and the ratio viral load per TCID50 of two H1N1 stains.

Stain	Viral load	TCID50	Viral load/TCID50
A/Shanghai/37T/2009 (H1N1)	1.59×10^9^	10^3.5^	4.97×10^5^
A/Shanghai/143T/2009 (H1N1)	5.50×10^8^	10^2.9^	6.88×10^5^

### Real-time neutralization test

**Figure 3:**
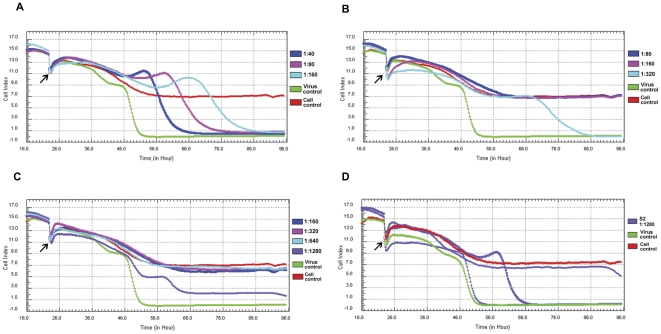
Typical results of real-time neutralization tests against SH37T. CPE kinetic patterns of MDCK cells inoculated with 100 TCID50 virus incubated with a series dilution of sera samples. Antibody titers were calculated as a function of CI with a sequential dilution series of S0 (A), S1 (B), and S2 (C). The curve was an average of three independent replicate wells. At the indicated time point the cells were inoculated with the serial serum-virus mixture. Three replicate wells of 1280-fold diluted S2 treated cells showed different curves (D). Cell control: MDCK cells only; virus control: absence of test sera.

Since H1N1-induced CPE was accurately quantified using the RTCA system, we hypothesized that blocking or delaying CPE by specific neutralizing antibodies from sera could be quantitated using the same method. To explore this concept, the neutralization tests were performed for 21 sera triples using the RTCA System. Neutralization titers were reported as the reciprocal of the highest serum dilution that inhibited virus growth by 100%. MDCK cells on E-Plates were inoculated with serially diluted sera triples that were pre-incubated with SH37T. Initial serum dilutions of 1∶40 for S0, 1∶80 for S1, and 1∶160 for S2 were chosen because at higher serum concentrations virus replication in MDCK cells was clearly inhibited. A total of 100 TCID50 of virus per assay well was achieved to reach maximal cell death. H1N1-induced CPE was monitored in real-time using the RTCA system. The typical results are illustrated in Figure 3. The CI of S0-treated cells declined to 0 entirely, and the resulting curves appeared similar to virus controls (Figure 3A), indicated that the S0 dilution above 20-fold could not inhibit virus growth. However, for the S1 and S2 samples, cell death was not observed until the serum was diluted to 320- and 1280-fold, respectively, and the resulting curves appeared similar to control cells (Figure 3B, C). This indicated that the neutralizing antibody titers in S1 and S2 were 160 and 640, respectively. Interestingly, the average curve of 1280-fold diluted S2-treated cells exhibited difference with virus control (Figure 3C), as the three replicate wells showed distinctly different CI curves (Figure 3D); one started to decline at about 90 hours, whereas the other two had already declined to 0 by this time point. Among the three wells treated with 1280-fold diluted S2, virus in two wells was neutralized completely but the rest one was not. So the finally showed average curve actually resulted from a partial neutralization pattern which was different from those of virus control and complete neutralization wells.

### Comparison of the real-time NT and HI test

**Figure 4:**
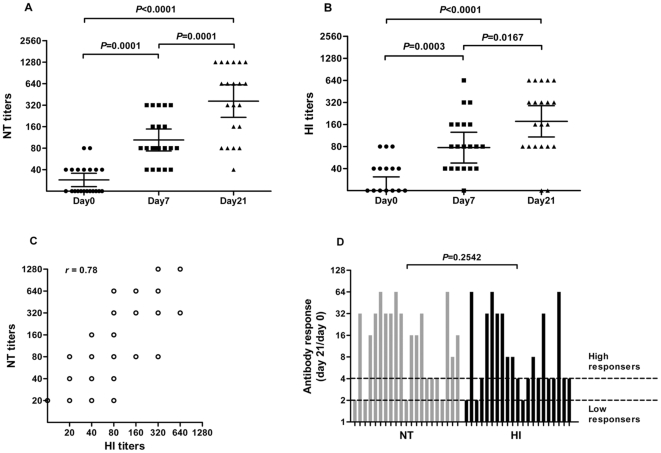
Antibody titers in 21 pre- and post-vaccination sera triples measured by real-time NT and HI tests. A, B) There was a statistically significant difference (*p*<0.01, Wilcoxon signed rank test) when the pre- and post-vaccination titers obtained by real-time NT (A) or HI test (B) were compared to each other. C) The NT and HI antibody titers correlated well (Spearman's correlation *r* = 0.78, *p*<0.01). D) Antibody titers on day 21 after vaccination relative to baseline (day 0). There was no significant difference (*p*>0.05, Wilcoxon signed rank test) in the results among the NT versus HI tests. Low responders: no increase above twofold; high responders: fourfold or more above baseline.

A total of 21 pre- and post-vaccination sera triples were collected and tested by real-time NT and HI tests (Table S1). Geometric mean pre- and post-vaccination titers (GMT), as determined by NT and HI tests, and the number of serum pairs exhibiting a significant increase in antibody titers (≥4-fold) were calculated (Table 2). The mean NT titer increase was 4-fold (range 1–16) at day 7 and 13-fold (range 2–64) at day 21 after vaccination, while the mean HI titer increase was 4-fold (range 1–16) and 9-fold (range 2–64). The rise in antibody levels between the pre- and post-vaccination sera is illustrated in Figure 4. Almost all of the 21 subjects exhibited significantly increased antibody titers to the H1N1 virus after vaccination, as determined by both the NT and HI test. When the pre- and post-vaccination titers obtained by HI or NT were compared to each other, there was a statistically significant difference (*p*<0.05, Wilcoxon signed rank test) (Figure 4A, B). The real-time NT and HI antibody titers showed a close correlation (Spearman's correlation coefficient, *r* = 0.78, *p*<0.01; Figure 4C). Furthermore, the magnitude of the antibody response to the vaccine was calculated as the maximum difference between the titer at day 21 and the baseline titer (day 0) for both the NT and HI test (Figure 4D). There was no difference in the mean antibody response measured by NT versus the HI test (*p*>0.05, Wilcoxon signed rank test). As a consequence, the NT assessed using the RTCA system was in good agreement with the HI test. This indicated that the RTCA system could be used to accurately quantify antibody titers.

**Table 2:** Geometric mean titers (GMT), titer ranges, and significant titer rises observed from 21 pre- and post-vaccination serum triples.

Test	GMT			Titer ranges			Number of significant antibody rises (%) (S2/S0 ≥4)
	S0	S1	S2	S0	S1	S2	
NT	29	104	365	20–80	40–320	40–1280	17(81%)
HI	20	77	177	<10–80	<10–640	20–640	18(86%)

## Discussion

With the emergence of the 2009 influenza pandemic, development of an effective vaccine became critical for the prevention and clearance of influenza virus infections. To evaluate seasonal or pandemic candidate vaccines, a sensitive, specific, and reproducible serological method is necessary to measure the antibody response elicited with a candidate vaccine against wild-type viruses. In this study, we developed a novel real-time method for measuring virus-induced CPE and evaluating the efficacy of neutralizing antibodies using electronic impedance biosensor-based RTCA technology. The RTCA system is based on integrated microelectronic sensor arrays, is non-invasive, and provides kinetic data regarding the dynamic nature of the cellular response to certain challenges, such as drug treatments [20], growth factor stimulation [21], analysis of cytotoxic composite components [22], and virus-induced cytopathogenesis [14].

In this report, this new assay was used to monitor cytolysis induced by H1N1 viral proliferation. Virus-induced CPE kinetic curves could be used to differentiate between different infectious amounts of the same strain as well as to distinguish the infection kinetics of different H1N1 strains. The two strains, SH37T and SH143T, were isolated respectively from two hospitalized patients in SHAPHC received diagnostic of 2009 H1N1 infection at May 2009. While the clinical patterns were essentially similar, these strains revealed different kinetics of viral infection. The time points for both the onset of CPE and complete cell death of SH143T-infected cells were delayed about 10 hours compared with those of SH37T-infected cells. Because an identical viral load was used, SH37T appeared to have a greater infectivity than SH143T. Therefore, the RTCA system can provide an indicator of virus capacity of replication and infectivity on MDCK cell culture. It was deserved to utilize complementary genetic approaches to define the role of specific viral genes and proteins underlying the phenotypes. Using real-time analysis, Ying Fang *et al.* [14] generated a mathematical model simulating the CI response to different virus species, through which the virus replication doubling time, virus number threshold for host cell death, and average time to cell death was determined. Meanwhile, the RTCA assay could directly distinguish different strains of the same virus on the basis of our results. For quantitation of virulence, it is necessary to compare different viral stains, determine the number of infectious units required to produce the specific endpoint [18]. Taken together, it is indicated that the RTCA system may provide a feasible *in vitro* format for assessment of virulence by monitoring CPE kinetics in future investigations of the influenza virus. It may further help to elucidate the relationship between genetic alterations and virulent variations for virological surveillance of influenza.

Since virus-induced CPE could be quantitatively monitored using the RTCA system, we also performed a real-time neutralization assay for measuring H1N1-specific antibodies in human sera using the RTCA system. Since the HI test is still used as a standard for epidemiological and immunological studies, as well as for measuring the efficacy of influenza vaccines and potency of neutralizing antibodies, this new assay was evaluated against the standard HI test on a panel of sera collected from adult donors before and after immunization with the H1N1 vaccine. Both assays showed an obvious increase in neutralizing antibodies in the test sera at 3 weeks post-vaccination, and there was a good agreement between the HI and NT antibody titers. However, higher antibody titers were detected using the RTCA-based NT method, indicating the higher sensitivity of this assay. This may have been due to the difference between the CPE-based and HA-based assays, which had been observed in previous reports [23], [24]. Such functional quantitation may provide a valuable platform for serological diagnosis, immunological study, or evaluation of vaccines for influenza.

An HI titer of 1∶40 is commonly recognized as representing protective immunity. Our results revealed 30% of the HI titers in the pre-vaccination sera were equal to 1∶40, and two were even higher (data not shown). The first H1N1 case was identified in May 2009 in Shanghai, and population-wide vaccination followed in October 2009. Hence, widespread exposure to H1N1 in the society may have caused the higher antibody titers in the donors detected here. In addition, other reports have suggested that serum cross-reactive antibody responses to H1N1 occurred after vaccination with seasonal influenza vaccine and that this also existed in the population during the pre-pandemic period [25], [26], [27].

In many settings influenza is recognized as a major cause of disease and death worldwide, which is the first infectious disease with global surveillance. Not the 2009 Influenza A (H1N1) Virus but other concomitant seasonal or highly pathogenic avian influenza viruses have posed considerable threads to public health. Global surveillance and annual vaccination are both of the key strategies and measures for the prevention and control of influenza. Apart from the evaluation of vaccine immunogenicity, the RTCA assay we described here can be used as a fundamental tool in the virological surveillance of influenza.

Assays with 2009 Influenza A (H1N1) Virus and other seasonal influenza viruses are performed biosafety level-2 (BSL-2), whereas assays with replication competent wide-type H5N1 and other potentially pandemic viruses must be performed under biosafety level-3 (BSL-3) containment, requiring convenient and labor-saving assays to minimize the hands-on steps. Conventional virus quantification and neutralization tests for influenza viruses require several manipulations, which are laborious and rather slow. However, the RTCA assay provides data continuously and automatically in a remote manner after the initial virus inoculation, which could save on time and labor for assay development and also decrease the risk of aerosol generation. With the few hands-on manipulations required, this new format allows for label-free, high throughput, and automatic testing, and could easily be adapted to assays under BSL-3 conditions.

## Supporting Information

Table S1 **Antibody titers in 21 pre- and post-vaccination serum triples.**
(DOC)
