# Correction: Novel, Real-Time Cell Analysis for Measuring Viral Cytopathogenesis and the Efficacy of Neutralizing Antibodies to the 2009 Influenza A (H1N1) Virus

**DOI:** 10.1371/annotation/9f718228-cb10-444b-882b-5dc795e9d1e7

**Published:** 2012-07-12

**Authors:** Di Tian, Wanju Zhang, Jing He, Yi Liu, Zhigang Song, Zhitong Zhou, Min Zheng, Yunwen Hu

Author Min Zheng (minzheng@aceabio.com.cn) should also be listed as a corresponding author. 

