# Supplementary material for: Novel, Real-Time Cell Analysis for Measuring Viral Cytopathogenesis and the Efficacy of Neutralizing Antibodies to the 2009 Influenza A (H1N1) Virus
Source: PLoS One. 2012 Feb 20;7(2):e31965. doi: 10.1371/journal.pone.0031965 (PMC3282789; doi:10.1371/journal.pone.0031965)
Supplement: Table S1 — Antibody titers in 21 pre- and post-vaccination serum triples. (DOC) [file pone.0031965.s001.doc]

**Table S1. Antibody titers in 21 pre- and post-vaccination serum triples.**

| Subjects  no. | A/Shanghai/37T/2009 (H1N1) | | | | | | |
| --- | --- | --- | --- | --- | --- | --- | --- |
|  | NT Test | | |  | HI Test | | |
|  | S0 | S1 | S2 |  | S0 | S1 | S2 |
| 1 | 40 | 40 | 80 |  | 40 | 80 | 80 |
| 2 | 20 | 320 | 640 |  | <10 | 320 | 320 |
| 3 | 80 | 80 | 160 |  | 40 | 40 | 80 |
| 4 | 40 | 40 | 640 |  | 40 | 40 | 160 |
| 5 | 40 | 160 | 1280 |  | 20 | 80 | 640 |
| 6 | 20 | 320 | 1280 |  | <10 | 160 | 320 |
| 7 | 40 | 80 | 1280 |  | 20 | 80 | 640 |
| 8 | 40 | 80 | 1280 |  | 20 | 80 | 640 |
| 9 | 20 | 320 | 1280 |  | 80 | 320 | 640 |
| 10 | 20 | 160 | 640 |  | 40 | 80 | 320 |
| 11 | 20 | 40 | 40 |  | 20 | 40 | 80 |
| 12 | 20 | 80 | 320 |  | 80 | 80 | 160 |
| 13 | 40 | 80 | 640 |  | 20 | 40 | 80 |
| 14 | 20 | 320 | 640 |  | 40 | 160 | 320 |
| 15 | 20 | 80 | 80 |  | 80 | 160 | 320 |
| 16 | 80 | 80 | 320 |  | <10 | 160 | 160 |
| 17 | 20 | 40 | 80 |  | <10 | 20 | 20 |
| 18 | 40 | 40 | 80 |  | <10 | <10 | 20 |
| 19 | 20 | 320 | 1280 |  | 10 | 640 | 640 |
| 20 | 20 | 80 | 160 |  | 20 | 40 | 80 |
| 21 | 20 | 160 | 320 |  | 20 | 40 | 80 |

<10: not detected
